# A Novel Reconstruction Approach After Skin Cancer Ablation Using Lateral Arm Free Flap: A Serial Case Report

**DOI:** 10.3390/medicina60122082

**Published:** 2024-12-19

**Authors:** Soyeon Jung, Seungjun Lee, Seokchan Eun

**Affiliations:** 1 Department of Plastic and Reconstructive Surgery, Hallym University Dongtan Sacred Heart Hospital, Hallym University College of Medicine, Hwaseong 18450, Republic of Korea; ps.soyeon.jung@gmail.com; 2 Department of Plastic and Reconstructive Surgery, Seoul National University Bundang Hospital, Seoul National University College of Medicine, Seongnam 13620, Republic of Korea

**Keywords:** free flap, lateral arm flap, face defects, temple, skin cancer

## Abstract

*Background and Objectives*: The lateral arm flap has been a very useful choice for the reconstruction of small to medium-sized defects, such as in the hands, extremities, and oral head and neck area. Its versatile characteristics and surgical feasibility allow this flap to be widely applied, but its reconstructive potential in the facial subunit after tumor ablation procedures has never been reported. In this study, we aimed to utilize the advantages of this flap to carry out facial temple subunit defect reconstruction. *Materials and Methods*: Between 2020 and 2023, 12 patients underwent temple reconstruction with lateral arm free flaps after wide malignant tumor excisions. There were seven women and five men, and the mean patient age was 60.6 years. Among the patients with cancer, six had squamous cell carcinoma, five had basal cell carcinoma, and one had myxofibrosarcoma. All flaps were elevated under general anesthesia. Alprostadil (PGE1, Eglandin^®^, Mitsubishi Tanabe Korea, Seoul, Republic of Korea) was administered postoperatively. *Results*: All flaps were the fasciocutaneous type, with sizes that varied from 3 cm × 4 cm to 5 cm × 7 cm (average size: 22.7 cm^2^). The average pedicle length was 6.1 cm. The versatility of the lateral arm flap enabled successful coverage in all cases, with no specific complications. Good functional outcomes and good ranges of motion in the donor arms were observed after surgery. *Conclusions*: The authors successfully verified the advantages of lateral arm flaps in the treatment of medium-sized facial temple subunit defects.

## 1. Introduction

The incidence of facial skin cancer has been increasing with age and changes in the environment [1]. Ablative surgery for skin cancer is also increasing to respond to the prevalence of skin cancer. Local flaps and skin grafting play a key role in covering the defect caused by facial skin cancer resection [2]. With the development of microsurgery, a variety of free flap techniques have been introduced to reconstruct the defects caused by facial skin cancer ablation. After facial skin cancer surgery, it is important to choose an appropriate flap for the patient’s specific needs, where both the requirements of the recipient site and the characteristics of the donor site are considered. The lateral arm free flap is a very widely used free flap due to its various advantages and its applicability. Although it has a relatively short pedicle length, it has several advantages, such as its constant vascular anatomy, and its dissection is not difficult [3,4,5]. It is an especially good option for the reconstruction of small to medium-sized defects on the hands, forearms, elbows, feet, and lower legs and in the head and neck region [6,7,8,9,10]. Easy dissection, minimal donor site morbidity, and a constant vascular anatomy with a long pedicle are the advantages of this flap [11,12]. However, there have been no reports of face-specific subunit reconstruction after malignant tumor ablation. In the present study, we evaluated patients that successfully underwent reconstruction using lateral arm free flaps after temple skin cancer surgery.

## 2. Materials and Methods

**Figure 1:**
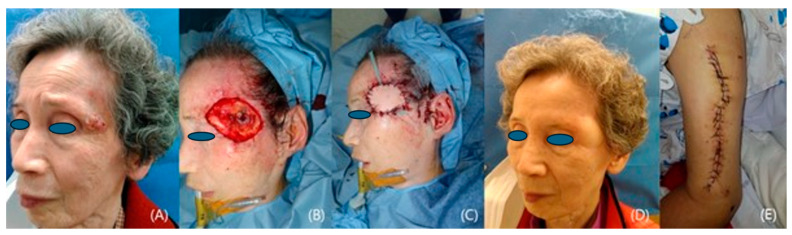
A case of a patient with left temple squamous cell carcinoma. (**A**) A preoperative photograph. (**B**) The 4 cm × 6 cm defect after wide excision. (**C**) A photo taken immediately after reconstruction with a lateral arm flap. (**D**) A photo taken 18 months after the operation. (**E**) The primarily closed donor site.

**Figure 2:**
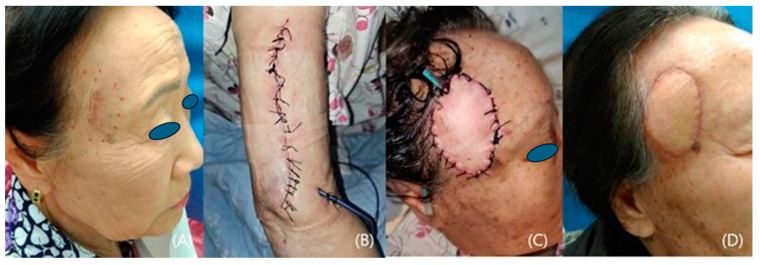
A case of a patient with right temple squamous cell carcinoma. (**A**) A preoperative photo. (**B**) Photos taken immediately after flap donor site closure and (**C**) reconstruction with a 5 cm × 3 cm lateral arm flap. (**D**) A photo taken 8 months after the operation.

**Figure 3:**
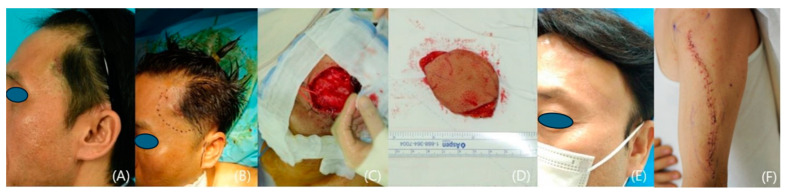
A case of a patient with left temple myxofibrosarcoma. (**A**) A preoperative photo. (**B**) A photo showing skin markings for a wide excision. (**C**) A photo taken immediately after tumor excision and recipient preparation. (**D**) The harvested 4 cm × 6 cm lateral arm flap. (**E**) A photo taken 22 months after the operation. (**F**) A photograph of the donor site, one month after the surgery.

**Figure 4:**
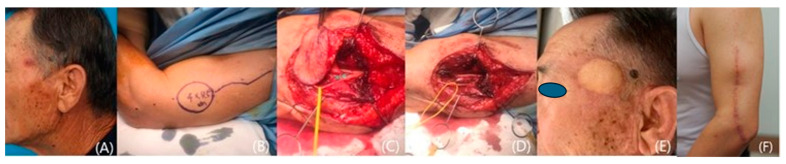
A case of a patient with left temple squamous cell carcinoma. (**A**) A preoperative photo. (**B**) The design of the 4 cm × 4.5 cm lateral arm flap. (**C**) Only the septal branch was included in the flap. (**D**) Preservation of the posterior antebrachial cutaneous nerve (PABCN). (**E**) A photo taken 19 months after the operation. (**F**) A postoperative view of the donor site, 19 months after the surgery.

**Figure 5:**
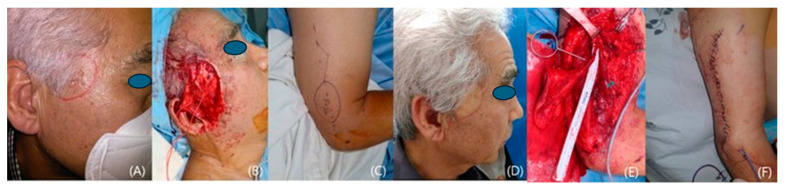
A case of a patient with right temple basal cell carcinoma. (**A**) A preoperative photo. (**B**) A photo taken after tumor resection and the preparation of the recipient superficial temporal vessels. (**C**) The design of the 3 cm × 4 cm lateral arm flap. (**D**) A photo taken 18 months after the operation (**E**) An intraoperative view of the harvested lateral arm flap preserving the septal branch of the PABCN with a sufficient vascular pedicle length. (**F**) A photograph of the donor site primarily closed.

We included 12 patients who underwent reconstruction with fasciocutaneous lateral arm free flaps from 2020 to 2023 at a single institution. Our study comprised 7 women and 5 men with a mean age of 60.6 years (range: 47–81). All the patients suffered from skin cancer that had developed on the temple (Figure 1, Figure 2, Figure 3, Figure 4 and Figure 5). Among the patients with cancer, six had squamous cell carcinoma (Figure 1, Figure 2 and Figure 4), five had basal cell carcinoma (Figure 5), and one had myxofibrosarcoma (Figure 3). We performed an institutional review board-approved retrospective review of our database to identify patient pools. This study was conducted according to the guidelines of the Declaration of Helsinki and was approved by the Institutional Review Board of Seoul National University (B-2402-880-103). Demographic and operative data pertaining to all patients are presented in Table 1.

### Surgical Technique

The patients were placed in the supine position, and a flap was harvested from an upper limb on an arm table with mild elbow flexion. No pneumatic or Esmark rubber tourniquets were applied. The flap was outlined on the distal third of the lateral aspect of the arm. The axis of the skin island of the flap was centered with a line drawn from the deltoid insertion to the lateral epicondyle, which corresponded to the lateral intermuscular septum. The flap width did not exceed 6 cm to allow for the skin closure of the donor defect (Figure 4B and Figure 5C). Flap elevation was possible from either the posterior or anterior directions. The flap was elevated deep into the muscular fascia over the triceps or brachialis, and was peeled until the septum was encountered.

This fascia was included in the flap to preserve vascularity. As the flap was elevated toward the septum, small muscular perforators were encountered and needed to be coagulated or ligated. The fascia in this region was slightly more attached to the muscles, and the flap could be elevated in the distal to proximal direction. Staying deeper than these vessels, the intermuscular septum was released to the level of the humeral periosteum and was raised, with the periosteum, from the humerus to the level of the deltoid insertion. As the dissection proceeded proximally, the distance between the vessels and the humerus increased and the dissection was easier. If the neurosensory flap needed to be harvested, only the septal branch was included in the flap, and the posterior antebrachial cutaneous nerve (PABCN) was preserved under loupe magnification (Figure 4C,D). At this stage, the radial nerve came into view proximally. Once an adequate pedicle length was obtained, the posterior radial collateral artery (PRCA) and venae comitantes were divided. After the flap was transferred, microanastomosis for arterial supply was carried out between the PRCA and the main trunk of the superficial temporal artery in most cases. On the other hand, the PRCA was connected to the frontal branch of the superficial temporal artery in four patients with a short pedicle. Venous anastomosis between the venae comitantes and superficial temporal vein was continued.

## 3. Results

There was no tumor recurrence. All flaps survived, provided stable coverage for the various defects, and produced satisfactory outcomes with good contours. Flap debulking surgery was not conducted. The dimensions of the lateral arm flaps ranged from 3 cm × 4 cm to 5 cm × 7 cm (average size: 22.7 cm^2^). The average pedicle length was 6.1 cm. All patients whose donor sites underwent primary closure attained a full range of motion in their donor arm during the follow-up period, without radial nerve injury (Figure 1E and Figure 5F). A small sensory deficit on the dorsolateral aspect of the forearm occurred in three patients. The posterior cutaneous nerve of the forearm was apparently preserved in the other cases. The donor site scar was tolerated well by all patients (Figure 3F and Figure 4F).

### 3.1. Serial Case Report

#### 3.1.1. Case 1

An 81-year-old female patient was diagnosed with a 2.2 cm × 1.3 cm squamous cell carcinoma on her left temple. A wide excision was used to ensure complete tumor removal, and the temporal branch of the facial nerve was successfully preserved. A 4 cm × 6 cm lateral arm free flap was harvested and transferred to the defect area, and it provided ample tissue coverage. The flap healed uneventfully with no tumor recurrence (Figure 1).

#### 3.1.2. Case 2

A 78-year-old woman was referred to our clinic with squamous cell cancer in her right temporal area. She had already undergone an excisional biopsy of a 2 cm × 1 cm protruding lesion at another hospital, and there was a linear scar on her temple. We planned her reconstruction using a lateral arm free flap from her right upper arm, with wide lesion excision (1 cm tumor-free resection margin). After the extensive excision of the tumor, the recipient’s superficial temporal artery and vein were prepared. After the flap was transferred, the superficial temporal vessels were anastomosed to the posterior radial collateral artery and its vena comitantes, and the operation was completed after the flap was inset. Seven months after the operation, the patient was relatively satisfied with the surgical scars at both the donor and recipient sites (Figure 2).

#### 3.1.3. Case 3

A 52-year-old male with a palpable mass in his left temporal area was referred to a plastic surgery clinic. A punch biopsy showed that the mass was myxofibrosarcoma. We widely excised the tumor with a 1~1.5 cm surgical safety margin. Fresh frozen sections were found to be negative, with free margins. A lateral arm fasciocutaneous flap was elevated and transferred to the defect. Microanastomosis was carried out using superficial temporal vessels as recipients. The flap healed uneventfully with no tumor recurrence (Figure 3).

#### 3.1.4. Case 4

A 71-year-old man with a squamous cell carcinoma on his left temple visited our clinic. The cancer measured 1.5 cm × 2.5 cm, and preoperative MRI showed invasion into the subcutaneous tissue close to the superficial temporal fascia. A radical resection was performed, including the superficial temporal fascia with a 1 cm surgical safety margin. Fresh frozen sections showed free margins. We reconstructed the defect using a left fasciocutaneous lateral arm free flap. The flap and the donor site healed uneventfully. Eleven months later, there was no tumor recurrence, and the flap showed excellent results with good contours. However, left forehead hemi-palsy was noticed, owing to the resection of the frontal branch of the facial nerve (Figure 4).

#### 3.1.5. Case 5

A 53-year-old man visited our clinic with basal cell cancer on his right temple. We widely excised the tumor with a 1 cm surgical safety margin and successfully preserved the temporal branches of the facial nerve. We harvested a 3 cm × 4 cm lateral arm flap and prepared the superficial temporal artery and vein underneath the tumor. The flap healed uneventfully, with no tumor recurrence (Figure 5).

## 4. Discussion

Ultraviolet (UV) radiation from the sun is the leading cause of skin cancer. Cumulative sun exposure mainly causes basal cell and squamous cell skin cancer on the face, including the temple area [13]. We tried to completely remove skin cancer, along with a pre-determined margin of healthy-looking skin around its edges. The safety margin was determined based on the size and thickness of the tumor, along with other characteristics such as ulceration. Samsanavičius et al. reported that they performed sentinel lymph node biopsies (SLNBs) in low- and high-risk cutaneous squamous cell carcinoma patients, but we did not perform SLNBs in these patients because lymph node metastasis in the temple area is reported very rarely in the literature [14,15].

Facial defects arising from tumor surgery can lead to aesthetic and functional deficits. Reconstruction in the facial temple subunit region requires a pliable flap with a good color match and the integration of the flap into the recipient site for optimal contours [16]. Local flaps can be considered as a primary choice of reconstruction for facial defects. It is also beneficial to match a good skin color and texture. Nevertheless, it is frequently necessary to choose another option for defects requiring a wider size of flap. Free flap surgery has been widely performed for the reconstruction of various defects for several reasons. High success rates, fast healing, and good functional and esthetic results have popularized the application of free flaps after tumor ablation procedures. Several types of free flaps are available in the body, and surgeons can choose from a wide variety of flaps.

For many years, the radial forearm flap was the fasciocutaneous flap of choice for reconstruction in the head and neck region. This flap provides a very thin skin paddle, which is ideal for intraoral reconstruction and all areas needing very pliable flaps [17]. However, in recent years, donor site morbidity has been considered as important as recipient site morbidity [18]. When a radial forearm flap is harvested, a major artery supplying blood to the hand has to be sacrificed, which might compromise the vascular supply to the hand. Moreover, the scar from a radial forearm flap is difficult to cover, and an additional skin graft may increase the scar width, causing abnormal pigmentation and depression [19]. The anterolateral thigh (ALT) or thoracodorsal artery perforator (TDAP) flap is a medium-sized or large flap with particularly long pedicles; however, in the temple subunit, large flaps and long pedicles are not needed. Also, these flaps have anatomical variations and are not stable, which subsequently increases the risk of complications, including the partial necrosis of the flap [19,20]. For this reason, the lateral arm flap is considered a more suitable alternative. It has a constant vascular anatomy, and there is no damage to the vascular supply of the hand because the flap contains a non-essential terminal branch of the profunda brachii artery [21,22]. The lateral arm is a donor site with better color matching than the radial, fibula, thigh, and latissimus regions. A flap may be harvested in a bloodless field using a tourniquet. Especially in oncologic and multi-morbid patients, it is important to reduce the operating time. This is possible with a two-team approach, with one team raising the flap and the other team resecting the tumor and preparing the recipient site [23,24].

There are only a few limitations restricting the use of the lateral arm flap. The amount of tissue available restricts its use to the reconstruction of small to moderate-sized defects. However, for temple subunit reconstruction, the lateral arm flap is a perfect choice because a large skin paddle is not needed. For the reconstruction of large skin defects, other fasciocutaneous flaps like the ALT or TDAP flaps are needed [25]. In addition, some local flaps can be employed to reconstruct the small defect caused by cancer ablation [26]. When considering the surgical procedure of free tissue transfer, the relatively small diameters and short pedicle lengths of the vessels are another disadvantage of this flap. However, for temple reconstruction, they are long enough to reach the recipient’s superficial temporal vessels and are an adequate size for microanastomosis [27].

Postoperative sensory disturbances in the dorsolateral forearm area might be disturbing to patients but will eventually improve with time [28]. For better sensory preservation, surgeons can isolate the septal branch of the posterior antebrachial cutaneous nerve in the flap and maintain the main trunk within a few centimeters of the proximal lateral epicondyle to prevent permanent sensory loss after surgery [29]. In addition, a functional assessment should be conducted to evaluate the morbidity of the donor site. A lack of assessment is another limitation of this case series. In contrast, the reconstruction of the temporal branch of the facial nerve is sometimes required after cancer resection, especially in cases of squamous cell cancer or melanoma. If the cancer is adjacent to this facial motor nerve, there is no choice but to perform an en bloc excision that includes the nerve to prevent perineural invasion. In this case, the posterior cutaneous nerve of the lateral arm may be used as a vascularized nerve graft with a lateral arm free flap (Figure 5E). However, this has not been reported. Further precise anatomical and clinical research is required.

## 5. Conclusions

A variety of flaps are available, and ultimate success depends on the selection of the optimal flap. Easy and quick dissection, design variability, and potential sensory innervation make the lateral arm flap superior to some other fasciocutaneous flaps. Thus, satisfactory outcomes with lateral arm flap reconstruction can be expected, and we find that the lateral arm flap is very reliable and versatile in the reconstruction of facial temple subunit defects. Further studies with a larger series of cases should be conducted to confirm the utility and efficacy of flap application.

## Figures and Tables

**Table 1:** Patients’ demographics.

No.	Sex	Age	Area	Cancer Type	Flap Size	Pedicle Length	Recipient Vessels	Follow-Up Period, mo	Complications
1	F	58	Left temple	Basal cell carcinoma	4 × 5 cm^2^	6 cm	Left STA and STV *	15	None
2	M	56	Left temple	Squamous cell carcinoma	3 × 4 cm^2^	5 cm	Left STA and STV, Fr ^§^	32	Forehead hemipalsy
3	M	41	Right temple	Basal cell carcinoma	4 × 6 cm^2^	6 cm	Right STA and STV	24	None
4	M	70	Left temple	Squamous cell carcinoma	4 × 4.5 cm^2^	6 cm	Left STA and STV	19	Forehead hemipalsy
5	F	56	Right temple	Basal cell carcinoma	5 × 6 cm^2^	5 cm	Right STA and STV, Fr	26	None
6	F	78	Right temple	Basal cell carcinoma	4 × 5 cm^2^	6 cm	Right STA and STV	8	None
7	M	59	Left temple	Basal cell carcinoma	4 × 6 cm^2^	6 cm	Left STA and STV	22	None
8	F	67	Left temple	Basal cell carcinoma	5 × 7 cm^2^	5 cm	Left STA and STV	38	None
9	F	52	Right temple	Basal cell carcinoma	3 × 5 cm^2^	6 cm	Right STA and STV	26	None
10	F	58	Right temple	Squamous cell carcinoma	4 × 5 cm^2^	5 cm	Right STA and STV, Fr	28	Forehead hemipalsy
11	M	46	Left temple	Basal cell carcinoma	5 × 4 cm^2^	7 cm	Left STA and STV	18	None
12	F	70	Right temple	Basal cell carcinoma	3 × 4 cm^2^	6 cm	Left STA and STV	25	None

* STA and STV: superficial temporal artery and vein; § Fr: Frontal branch of superficial temporal artery.

## Data Availability

Data are contained within the article.
